# Manganese transport is essential for N_2_‐fixation by *Rhizobium leguminosarum* in bacteroids from galegoid but not phaseoloid nodules

**DOI:** 10.1111/1462-2920.13773

**Published:** 2017-05-30

**Authors:** Graham Hood, Vinoy Ramachandran, Alison K. East, J. Allan Downie, Philip S. Poole

**Affiliations:** ^1^ Department of Molecular Microbiology John Innes Centre Norwich Research Park Norwich NR4 7UH UK; ^2^ Department of Plant Sciences University of Oxford South Parks Road Oxford OX1 3RB UK

## Abstract

*Rhizobium leguminosarum* has two high‐affinity Mn^2+^ transport systems encoded by *sitABCD* and *mntH*. In symbiosis, *sitABCD* and *mntH* were expressed throughout nodules and also strongly induced in Mn^2+^‐limited cultures of free‐living cells. Growth of a *sitA mntH* double mutant was severely reduced under Mn^2+^ limitation and *sitA* and *mntH* single mutants were more sensitive to oxidative stress. The double *sitA mntH* mutant of *R. leguminosarum* was unable to fix nitrogen (Fix^‐^) with legumes belonging to the galegoid clade (*Pisum sativum*, *Vicia faba* and *Vicia hirsuta*). The presence of infection thread‐like structures and sparsely‐packed plant cells in nodules suggest that bacteroid development was blocked, either at a late stage of infection thread progression or during bacteroid‐release. In contrast, a double *sitA mntH* mutant was Fix^+^ on common bean (*Phaseoli vulgaris*), a member of the phaseoloid clade of legumes, indicating a host‐specific symbiotic requirement for Mn^2+^ transport.

## Introduction

The nitrogen (N_2_)‐fixing symbioses between rhizobia and legumes are a subject of intense study. To achieve effective symbioses, rhizobia must first initiate the formation of specialist organs, known as nodules, on the roots of legumes. Rhizobia then descend through the plant‐made infection threads and colonise nodules. The bacteria are engulfed by plant cells and are surrounded by a plant‐derived membrane, where they enlarge and differentiate into N_2_‐fixing bacteroids. These bacteroids and surrounding plant membrane are analogous to organelles and are called symbiosomes. It is the bacteroids that reduce N_2_ to ammonia (NH_3_) by a process known as N_2_ fixation (Terpolilli *et al*., 2012; Udvardi and Poole, 2013).

Some of the best‐characterised and agriculturally important legumes e.g. pea (*Pisum sativum*), alfalfa (*Medicago sativa*), broad bean (*Vicia faba*) and clovers (*Trifolium* species) belong to the galegoid clade. Two characteristics of galegoid legumes are their indeterminate nodules (Ferguson *et al*., 2010) and the presence in their genomes of genes encoding nodule‐specific cysteine‐rich (NCR) peptides (Mergaert *et al*., 2006; Van de Velde *et al*., 2010; Kondorosi *et al*., 2013). These NCR peptides, which show similarities to antimicrobial peptides, are responsible for the enlarged pleomorphic shapes of bacteroids, their chromosomal endoreduplication, altered membrane integrity and terminal differentiation (Oono *et al*., 2009; Karunakaran *et al*., 2010; Haag *et al*., 2013, 2011). In contrast to galegoid legumes, legumes belonging to the phaseoloid clade e.g. soybean (*Glycine max*) and common bean (*Phaseolus vulgaris*), form determinate nodules and lack NCR peptides. As a result, the bacteroids of these legumes are non‐swollen, do not endoreduplicate, do not have altered membrane permeability and are able to regrow outside the nodule (Mergaert *et al*., 2006; Kondorosi *et al*., 2013).

Microarray analyses of *Rhizobium leguminosarum* bv. viciae 3841 (Rlv3841) isolated from the nodules of the galegoid legume *P. sativum*, furthered our understanding of the fundamental differences between free‐living bacteria and swollen bacteroids (Karunakaran *et al*., 2009). Furthermore, the clustering of expression profiles at different time points revealed that rhizobia isolated from young nodules [harvested 7 days post inoculation (dpi)] had a significant number of up‐regulated‐genes that were not up‐regulated in mature bacteroids (15, 21 and 28 dpi) or in bacteria isolated from the rhizosphere surrounding the roots (Ramachandran *et al*., 2011). These highly up‐regulated‐genes are likely to be relevant to understanding processes integral to nodule colonisation and bacteroid development, and include genes encoding two putative Mn^2+^ transport systems, SitABCD (ABC transport system) and MntH (proton coupled system).

The requirement for Mn^2+^ uptake during symbiosis is not clear due to reported conflicting phenotypes when either *sitABCD* or *mntH* are mutated in rhizobia. For example, *Sinorhizobium meliloti sitABCD* mutants are compromised for growth under oxidative stress and have reduced rates of N_2_ fixation in symbiosis with the galegoid legume *M. sativa* (Chao *et al*., 2004; Davies and Walker, 2007a, 2007b). *Bradyrhizobium japonicum* lacks SitABCD and relies on MntH for Mn^2+^ uptake; however, deletion of *mntH* had no obvious effect on symbiosis with the phaseoloid legume *G. max* (Hohle and O'Brian, 2009). One explanation for this apparent difference in requirements for manganese may be that the requirement of Mn^2+^ uptake is dependent on whether the plant‐host is a galegoid or phaseoloid‐legume. To explore this further, SitABCD and MntH were studied in *R. leguminosarum* and their requirement for symbiosis examined in both galegoid and phaseoloid legumes.

## Results

### In Rlv3814 *sitA* and *mntH* are expressed throughout nodules and are regulated by Mur

**Figure 1:**
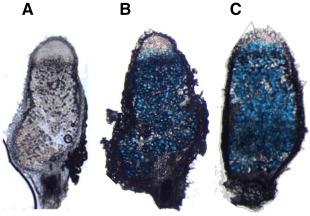
Nodules stained for GUS activity showing spatial expression of *sitAp*‐*gusA* or *mntHp*‐*gusA*. Nodules are colonised with Rlv3841 (A) or strains carrying either *sitAp*‐*gusA* (B) or *mntHp‐gusA* (C). Blue staining indicates the presence of GUS activity.

To verify the microarray data reported by Karunakaran *et al*., 2009, promoters of *sitA* (*sitAp*) and *mntH* (*mntHp*) were fused to a *gusA*‐reporter in the broad‐host‐range vector pJP2 (Prell *et al*., 2002). *P. sativum* was inoculated with Rlv3841 carrying either *sitAp*‐*gusA* or *mntHp*‐*gusA* and after three weeks, nodules were stained for β‐glucuronidase (GUS) activity. In both cases, GUS activity was detected throughout the nodule (Fig. 1).

**Figure 2:**
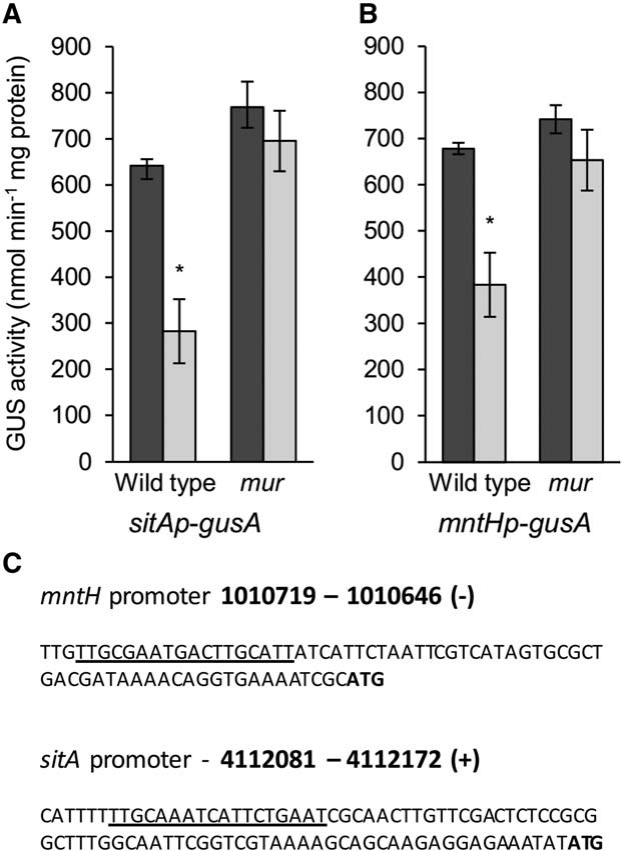
GusA activity in strains expressing *sitAp*‐*gusA* or *mntHp*‐*gusA* in response to Mn^2+^ and in the absence of Mur. GusA activity in Rlv3841 and the *mur* mutant carrying either *sitAp*‐*gusA* (A) or *mntHp*‐*gusA* (B). Measured in media limited (0.05 µM, dark bars) and not limited (0.9 µM, light bars) for MnSO_4_. Averaged from three independent experiments ± SEM. Statistical differences indicated by ‘*’ (*P* ≤ 0.05). (C). Putative promoter region of *mntH* and *sitABCD*, with the *mur* box underlined and the ATG start of each gene shown in bold. Absolute positions in the Rlv3841 genome of the transcribed strand are shown.

It was already known that in *R. leguminosarum* the *sitABCD* operon is regulated in response to Mn^2+^ by a Fur‐like repressor called Mur (manganese uptake regulator) (Diaz‐Mireles *et al*., 2004, 2005). The DNA‐binding site of Mur is well defined (Diaz‐Mireles *et al*., 2004; Rodionov *et al*., 2006) and can be found upstream of both *sitABCD* and *mntH* (Fig 2C). To confirm that *mntH* is regulated by Mur in response to Mn^2+^, expression of *sitA‐gusA* and *mntH‐gusA* constructs were compared in free‐living cells. As expected, higher levels of GUS activity were observed when the *sitAp*‐*gusA* strain was grown under Mn^2+^ limitation relative to when it was grown with excess MnSO_4_ (Fig. 2). The pattern of GUS activity was very similar for the *mntHp*‐*gusA* strain (Fig. 2), indicating that *mntH* is also regulated in response to Mn^2+^‐limitation. To see if this response is regulated by Mur, the *gusA*‐fusions were introduced into a *mur* mutant (Wexler *et al*., 2001). In the *mur* mutant, GUS activity with both *sitAp*‐*gusA* and *mntHp*‐*gusA* remained high regardless of the MnSO_4_ levels (Fig. 2). Therefore, both the *sitABCD* operon and *mntH* are regulated by the Mur‐repressor in response to Mn^2+^ levels in Rlv3841.

### Requirement of high affinity Mn^2+^ transport systems in response to Mn^2+^ limitation and oxidative stress

**Figure 3:**
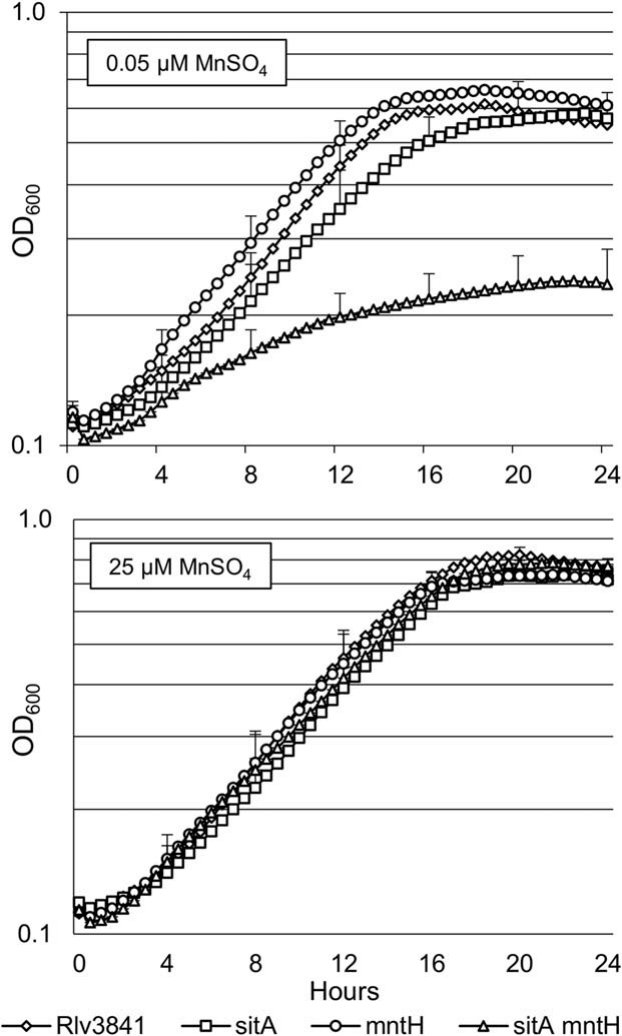
Growth curves of single and double *sitA mntH* mutants under Mn^2+^ limitation. Rlv3841 (diamonds), single mutants *sitA* (squares) and *mntH* (circles), and the double mutant *sitA mntH* (triangles) were grown in media limited (0.05 µM) or not limited (25 µM) for MnSO_4_. Averaged from three independent experiments. For clarity only plus SEM bars are shown at 4 h intervals.

To confirm SitABCD and MntH as the main Mn^2+^ transport systems in Rlv3841, growth of the single *sitA* and *mntH* mutants and the *sitA mntH* double mutant (Table 1) were tested under Mn^2+^ limitation. As expected, the single mutations by themselves did not cause a strong growth phenotype, although the *sitA* mutant did have a longer generation time relative to Rlv3841 under Mn^2+^ limitation (5.5 h c.f. 4.5 h) (Fig. 3). Growth of the double mutant however, was severely defective under Mn^2+^ limitation (Fig. 3), confirming SitABCD and MntH are the main transporters for Mn^2+^ in Rlv3841. Growth of the double mutant was restored by introducing a stable plasmid (pJP2) carrying *sitA* alone (along with its native promoter), indicating that the sitA‐pK19mob mutation is non‐polar (Supporting Information Fig. S1).

**Table 1:** Bacterial strains and plasmids used in this study.

Strain or plasmid	Description	Source or Reference
**Plasmid**		
pJET 1.2/Blunt	PCR product cloning vector; Ap^r^	Thermo Scientific
pK19mob	Integration plasmid; mob^+^; Km^r^	(Schafer et al., 1994)
pRK2013	Helper plasmid; mob^+^; Km^r^	(Ditta et al., 1980)
pJP2	Broad‐host‐range *gusA* transcriptional promoter probe vector; Tc^r^	(Prell et al., 2002)
pHP45Ω‐Spc	Vector carrying the Ω intersposon Sp^r^ cassette; Amp^r^ Sp^r^	(Fellay et al., 1987)
pHP45Ω‐Km	Vector carrying the Ω intersposon Km^r^ cassette; Amp^r^ Km^r^	(Fellay et al., 1987)
pJQ200SK	Suicide vector *sacB* gene; Gm^r^	(Quandt and Hynes, 1993)
pLMB452	Internal fragment of *sitA* cloned into pK19mob; Km^r^	This study
pLMB543	*mntH* cloned into pJET; Amp^r^	This study
pLMB544	*mntH*ΩSpc in pJET; Amp^r^	This study
pLMB546	*mntH*ΩSpc cloned into pJQ200SK; Gm^r^ Spc^r^	This study
pLMB592	Internal fragment of *oxyR* cloned into pK19mob; Km^r^	This study
pLMB597	*sitA* promoter cloned into pJP2; Tc^r^	This study
pLMB600	*mntH* promoter cloned into pJP2; Tc^r^	This study
pLMB679	*sitA* amplified from Rlp4292 and cloned into pJET; Amp^r^	This study
pLMB691	*sitA*ΩKm in pJET; Amp^r^ Km^r^	This study
pLMB694	*sitA*ΩKm cloned into pJQ200SK; Gm^r^ Km^r^	This study
pLMB766	*mntH* cloned into pJP2; Tc^r^	This study
pOPS0393	*sitA* along with its native promoter cloned into pJP2; Tc^r^	This study
pOPS0394	*sitABCD* operon along with its native promoter cloned into pJP2; Tc^r^	This study
**Strain**		This study
Rlv3841	Wild type *R. leguminosarum* bv. viciae; Str^r^ derivative of strain Rlv300; Str^r^	(Johnston and Beringer, 1975)
J325	*R. leguminosarum* bv. viciae J251; *mur*ΩSpc; Spc^r^	(Wexler et al., 2001)
RlvA34	*R. leguminosarum* bv. viciae formerly known as 8401/pRL1JI	(Downie et al., 1983)
Rlp4292	Derivative of field bean isolate 8002 with sym plasmid pRP2J1; Rif^r^	(Lamb et al., 1982)
LMB364	pLMB452 integrated into Rlv3841; *sitA*:pK19mob; Neo^r^	This study
LMB460	pLMB546 conjugated into Rlv3841; *mntH*ΩSpc; Spc^r^	This study
LMB466	*mntH*ΩSpc transduced from LMB460 into LMB364; *sitA*:pK19mob *mntH*ΩSpc; Neo^r^ Spc^r^	This study
LMB497	pLMB596 integrated into Rlv3841; *oxyR*:pK19mob; Neo^r^	This study
LMB498	pLMB597 conjugated into Rlv3841; *sitA*‐g*usA*; Tc^r^	This study
LMB505	pLMB600 conjugated into Rlv3841; *mntH*‐g*usA*; Tc^r^	This study
LMB511	pLMB597 conjugated into LMB497; *sitA*‐g*usA*; Tc^r^	This study
LMB512	pLMB600 conjugated into LMB497; *mntH*‐g*usA*; Tc^r^	This study
LMB525	*sitA*:pK19mob transduced from LMB364 into RlvA34; Neo^r^	This study
LMB526	*mntHΩSpc* transduced from LMB460 into RlvA34; Spc^r^	This study
LMB539	*sitA*:pK19mob transduced from LMB364 into LMB526; Neo^r^ Spc^r^	This study
LMB541	pLMB546 integrated into Rlp4292; *mntH*ΩSpc; Spc^r^	This study
LMB550	pLMB597 conjugated into J325; *sitA*‐g*usA*; Tc^r^	This study
LMB551	pLMB600 conjugated into J325; mntH‐gusA; Tc^r^	This study
LMB624	pLMB694 conjugated into 4292; *sitA*ΩKm; Neo^r^	This study
LMB630	pLMB694 conjugated into LMB541; *sitA*ΩKm *mntH*ΩSpc; Neo^r^ Spc^r^	This study
LMB683	pLMB766 (pJP2*mntH*) conjugated into LMB466 (*sitA*:pK19mob *mntH*ΩSpc); Neo^r^ Spc^r^ Tc^r^	This study
OPS0925	pOPS0393 (*sitA* along with its native promoter cloned into pJP2) conjugated into LMB466 (*sitA*:pK19mob *mntH*ΩSpc); Neo^r^ Spc^r^ Tc^r^	This study
OPS0926	pOPS0394 (*sitABCD* operon along with its native promoter cloned into pJP2) conjugated into LMB466 (*sitA*:pK19mob *mntH*ΩSpc); Neo^r^ Spc^r^ Tc^r^	This study

**Figure 4:**
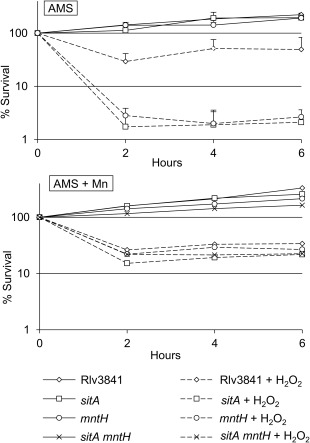
Sensitivity to H_2_O_2_. Rlv3841 (diamonds), single mutants *sitA* (squares), *mntH* (circles) and double mutant (crosses) were exposed to 0 mM (solid line) or 0.5 mM (broken line) H_2_O_2_ after growth in AMS or modified AMS glucose supplemented with 25 µM MnSO_4_. Survival (%) corresponds to number of colony forming units (CFU) relative to number of CFUs at time 0 h. Data from average of three independent experiments for ‘AMS’ or one experiment for ‘AMS supplemented with 25 µM MnSO_4_’. It was not possible to test the double mutant in the same way as it requires Mn^2+^‐rich medium for growth and high Mn^2+^ protects against oxidative stress.

Due to the presence of reactive oxygen species (ROS) in the infection threads of nodules (Santos *et al*., 2001; Rubio *et al*., 2004; Cardenas *et al*., 2008), the requirement for SitABCD and MntH was also examined under oxidative stress. Mn^2+^ is known for its critical role when cells are exposed to oxidative stress, acting both as a cofactor for protective enzymes (Santos *et al*., 2000; Kehres and Maguire, 2003; McEwan, 2009) and by suppressing Fenton's reaction by replacing Fe^2+^ in mononuclear enzymes (Anjem and Imlay, 2012; Imlay, 2013). To measure the sensitivity of the wild type and mutants to oxidative stress, cultures were exposed to hydrogen peroxide (H_2_O_2_), viable cells counted and it was found that the single mutants were hypersensitive to H_2_O_2_ (Fig. 4). It was not possible to test the double mutant in the same way as it requires Mn^2+^‐rich medium for growth and high Mn^2+^ protects against oxidative stress (Fig. 4). However, the phenotypes of the single mutants show that SitABCD and MntH help protect cells against oxidative stress. However, expression of *sitABCD* and *mntH* are not regulated in response to H_2_O_2_ and their expression is independent of the H_2_O_2_‐responsive regulator OxyR (Supporting Information Fig. S2).

### Requirement of Mn^2+^ transporters for symbiosis with P. sativum, V. faba and V. hirsuta (galegoid legumes)

**Figure 5:**
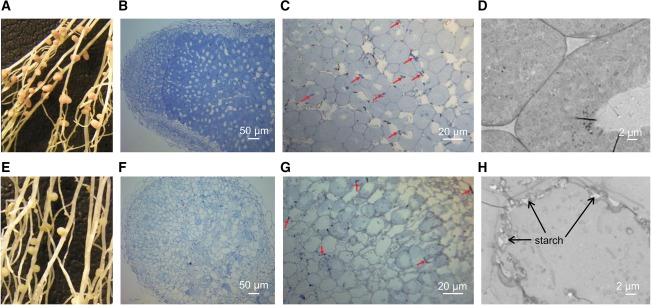
Symbiotic phenotypes of double mutant on *P. sativum*. Nodules colonised by Rlv3841 (A–D) or the *sitA mntH* double mutant (E–H) were harvested after three weeks. Images of whole nodules (A and E), nodule sections stained with toluidine blue (B, C, F and G) and electron micrographs (D and H) are shown. Red arrows (C and G) indicate infection thread‐like structures. Electron micrograph of nodule colonised by double mutant shows presence of starch.

**Figure 6:**
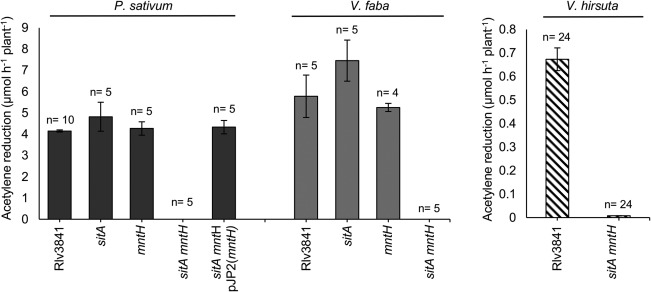
Rates of acetylene reduction for Rlv3841, single and double *sitA mntH* mutants on galegoid legumes *P. sativum*, *V. faba* and *V. hirsuta*. Measurements taken three weeks post inoculation. Averaged from four to ten plants or twenty‐four plants for *V. hirsuta* ± SEM.

**Figure 7:**
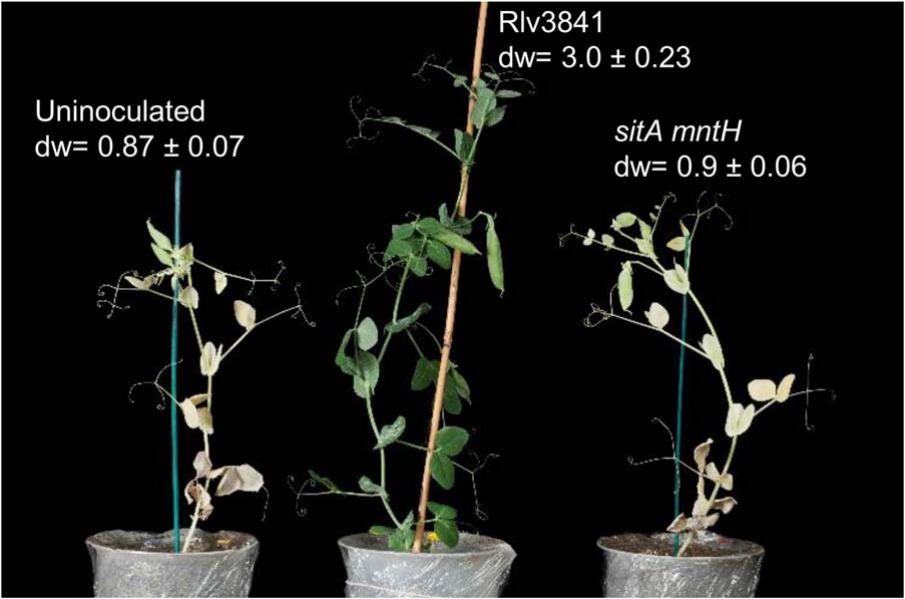
*P. sativum* inoculated with Rlv3841 or the *sitA mntH* double mutant, alongside an uninoculated control. Plants were grown for six weeks post inoculation. Shoot dry weights (g) denoted by ‘dw’ and averaged from ten plants ± SEM.

To test the requirement of SitABCD and MntH for nodule colonisation on galegoid legumes, the single and double mutants were inoculated onto *P*. sativum. Three weeks post inoculation, *P. sativum* nodules colonised by the single mutants were elongated, pink in colour and indistinguishable from nodules colonised by wild type Rlv3841 (Fig. 5a), whereas nodules induced by the double mutant were small, spherical, white in colour and typical of an ineffective symbiosis (Fig. 5e). Acetylene reduction assays indicated a lack of N_2_ fixation (Fig. 6) and after six weeks’ growth*, P. sativum* inoculated with the double mutant were indistinguishable from the uninoculated control (Fig 7.). N_2_ fixation was restored to the double mutant by complementing with *mntH* cloned in the stable plasmid pJP2 (Fig. 6), confirming the phenotype is due to Mn^2+^ transport.

Even though infection‐thread‐like structures could be seen (Figs. 5c and 5g) only a few of the nodule cells were infected by the double mutant and these cells were sparsely packed with bacteria (Figs. 5f and 5h), relative to the many densely‐packed plant cells containing Rlv3841 (Figs. 5b and 5d). Large starch granules were also present in nodules inoculated with the double mutant (Fig. 5h), indicative of a failed symbiosis (Udvardi and Poole, 2013).

To see if this Fix^‐^ phenotype extends to other galegoid legumes that fall within the host‐range of Rlv3841, the double mutant was tested on *V. faba* and *V. hirsuta*. As with *P. sativum*, a Fix^‐^ phenotype was observed for the double mutant on *V. faba* while no symbiotic defect was observed with the single mutants (Fig. 6.). *V*. *hirsuta* inoculated with the double mutant was also Fix^‐^ (Fig. 6). Thus on three separate galegoid legumes, Mn^2+^ uptake by Rlv3841 is essential for an effective N_2_‐fixing symbiosis.

### Requirement of Mn^2+^ transporters for symbiosis with the phaseoloid legume *P. vulgaris*

In light of the apparent lack of a requirement for Mn^2+^ transport for symbiotic N_2_ fixation with the phaseoloid legume *G. max* (Hohle and O'Brian, 2009), we decided to test the requirement for Mn^2+^ uptake in *R*. *leguminosarum* with the phaseoloid legume, *P. vulgaris*. The legume *P. vulgaris* is not a host of Rlv3841 but it is symbiotically compatible with *R. leguminosarum* bv. phaseoli 4292 (Rlp4292) (Lamb *et al*., 1982; Downie *et al*., 1983). Therefore, to check the requirement of Mn^2+^ uptake on *P. vulgaris, sitA* and *mntH* were mutated in Rlp4292. As a control, the two mutations were also introduced into *R. leguminosarum* bv. viciae A34 (RlvA34), a strain derived from Rlp4292 but possessing a different Sym plasmid, allowing it to nodulate and fix N_2_ on *P. sativum* (Lamb *et al*., 1982; Downie *et al*., 1983).

**Figure 8:**
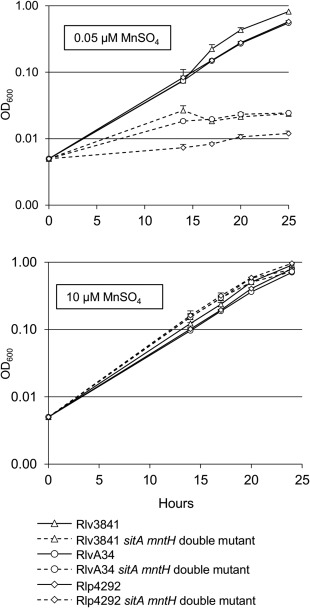
Growth curves for *sitA mntH* double mutants under Mn^2+^ limitation. Rlv3841 (triangles), RlvA34 (circles) and Rlp4292 (diamonds) wild type strains (solid line) were grown alongside their corresponding *sitA mntH* double mutant (broken line) in media limited (0.05 µM) and not limited (10 µM) for MnSO_4_. Averaged from three independent experiments.

**Figure 9:**
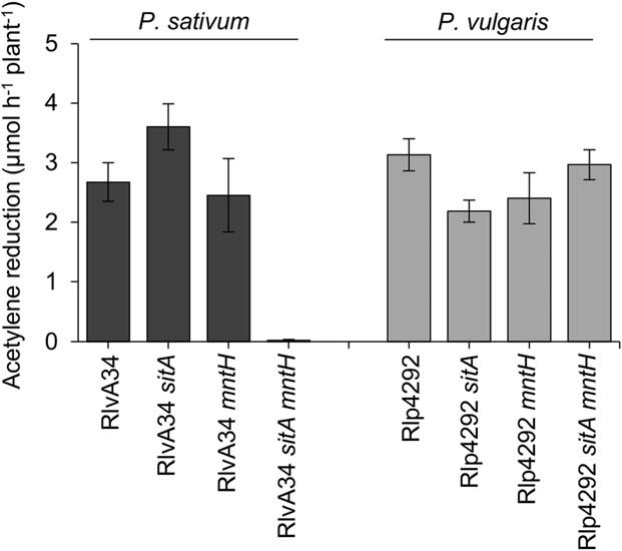
Rates of acetylene reduction for wild type and mutant strains of RlvA34 and Rlp4292 on *P. sativum* or *P. vulgaris*. *P. sativum* (galegoid) and *P. vulgaris* (phaseoloid) were harvested three and four weeks (respectively) post‐inoculation. Averaged from five plants ± SEM.

Similar to the Rlv3841 double mutant, growth of the Rlp4292 and RlvA34 double mutants were severely impaired under Mn^2+^ limitation (Fig. 8) confirming that in this different genetic background, SitABCD and MntH are the main transporters for Mn^2+^. When *P. sativum* was inoculated with the RlvA34 double mutant a Fix^‐^ phenotype was observed (Fig. 9), in agreement with the symbiotic phenotype of the Rlv3841 double mutant (Fig. 6). In contrast, when the phaseoloid legume *P. vulgaris* was inoculated with the Rlp4292 double mutant, the appearance of the nodules and the N_2_ fixation rates were indistinguishable from plants inoculated with Rlp4292 wild type (Fig. 9).

## Discussion

We show here that the Mn^2+^ transporters SitABCD and MntH are required by *R. leguminosarum* for symbiosis with legumes belonging to the galegoid clade i.e. *P. sativum*, *V. faba* and *V. hirsuta*. The presence of infection thread‐like structures and the sparsely‐packed plant cells suggest that bacteroid development of the double mutant is either blocked at a late stage of infection thread progression or during bacteroid‐release. A similar developmental phenotype was seen when *bacA* was mutated in *R. leguminosarum*, where BacA confers resistance against NCR peptides (Karunakaran *et al*., 2010; Haag *et al*., 2013). However, the *sitA mntH* double mutant showed no symbiotic phenotype on the phaseoloid legume *P. vulgaris*. Similarly, no symbiotic phenotype was observed when a *B. japonicum* mutant defective for Mn^2+^ uptake was used to inoculate the phaseoloid legume *G. max* (Hohle and O'Brian, 2009).

One simple explanation as to why no symbiotic phenotypes have been seen with phaseoloid legumes would be if there is much more Mn^2+^ available in the nodules of these legumes; consequently, high‐affinity Mn^2+^ transporters like SitABCD and MntH would become non‐essential. Consistent with this it has been reported that *P. vulgaris* grown symbiotically with rhizobia require high levels of Mn^2+^ for optimum growth (Pelaez *et al*., 2010). A second explanation could be that there is a lower concentration of ROS in the infection threads of phaseoloid relative to galegoid legumes. The presence of ROS in infection threads has been widely reported although we are not aware of direct comparison between galegoid and phaseoloid legumes (Santos *et al*., 2001; Rubio *et al*., 2004; Cardenas *et al*., 2008). The superoxide dismutase (SodA) in *S. meliloti* can use Mn^2+^ as a cofactor and its absence results in abnormal infection and senescent bacteroids (Santos *et al*., 1999, 2000). Although this seems similar to the phenotype of the *sitA mntH* double mutant, disruption of *sodA* in Rlv3841 was shown not to affect nodulation or N_2_ fixation on *P. sativum* (personal communication, Allan Downie). The symbiotic requirement of Mn^2+^ cannot therefore be completely attributed to SodA and instead may also reflect its ability to replace Fe^2+^ as a cofactor under oxidative stress (Anjem *et al*., 2009). Alternatively, the requirement of Mn^2+^ may not be restricted to oxidative stress resistance as some bacteria utilise the metal as a cofactor for enzymes central to metabolism (Eyzaguir *et al*., 1973; Hohle and O'Brian, 2012).

Mn^2+^ transporters, in particular SitABCD, have also been shown to be important for *S. meliloti* 1021 when colonising the nodules formed on the galegoid legume *M. sativa* (Chao *et al*., 2004; Davies and Walker, 2007a). Interestingly, the symbiotic phenotype of the *R. leguminosarum* double mutant is more severe than that of the *S. meliloti sitA* mutant. For example, when *M. sativa* was inoculated with the *sitA* mutant a mixture of small‐white and intermediate‐sized nodules were observed (Davies and Walker, 2007a) in contrast to the homogenous small‐white nodules initiated by the *R. leguminosarum* double mutant on all three galegoid legumes tested. In addition to this, mutation of *sitA* in *S. meliloti* only caused a ∼50–75% decrease in acetylene reduction whereas no acetylene reduction could be detected for the *R. leguminosarum* double mutant. A possible explanation for this discrepancy is that *S. meliloti* 1021 encodes another Mn^2+^ transporter. Indeed, it has been reported that the *S. meliloti* genome contains an uncharacterised gene (locus tag SMa115) that encodes a putative Nramp transporter that shares 26% amino acid identity with MntH from *E. coli* (Patzer and Hantke, 2001; Platero *et al*., 2007). Furthermore, this might also explain why mutation of *sitB* and *sitD* in *S. meliloti* strain 242 caused no symbiotic phenotype (Platero *et al*., 2003). In contrast to *R. leguminosarum* however, disruption of *sitABCD* alone in strain 242 caused a strong growth phenotype under Mn^2+^ limitation (Platero *et al*., 2003; Diaz‐Mireles *et al*., 2004), suggesting that SitABCD is the major Mn^2+^ transporter in *S. meliloti* under free‐living conditions.

An intriguing possibility is the presence of NCR peptides in galegoid legumes and their absence in phaseoloid legumes alter the susceptibility of rhizobia to oxidative stress. Antimicrobial peptides like NCR peptides have been shown to stimulate HO^.^ Formation via Fenton's reaction by inflicting damage on Fe‐S clusters (Kohanski *et al*., 2007). If this is the case, there would be a demand for Mn^2+^ to supress Fenton's reaction by replacing Fe^2+^ as a cofactor wherever possible (Anjem and Imlay, 2012). NCR peptides are also known to increase membrane permeability of bacteroids, which may lead to the leakage of cellular content (Galvez *et al*., 1991; Okereke and Montville, 1992; Maftah *et al*., 1993; Matsuzaki *et al*., 1997; Xu *et al*., 1999; Brogden, 2005; Bolintineanu *et al*., 2010). It is feasible therefore that transporters like SitABCD and MntH would become essential for retrieving the metal ions that are lost on exposure to NCR peptide. Membrane damage would also disrupt the proton motive force (pmf) of the membrane, conservation of which is critical to a range of divalent metal transporters (Karlinsey *et al*., 2010). In phaseoloid legumes therefore, the presence of active divalent metal transporters with a low affinity for Mn^2+^ may be able to compensate for the loss of SitABCD and MntH. In galegoid legumes however, where the functionality of these low affinity transporters may be compromised by the disruption of pmf and the rate of Mn^2+^ uptake may be insufficient to compensate for the loss of SitABCD and MntH.

For plant‐infecting bacteria this demonstrates how a bacterium's requirement for metal transporters depends on the host plant. The requirement of an Mg^2+^ channel by *R. leguminosarum* for N_2_ fixation on specific legumes is another example of how the plant‐host dictates the requirement for the transporters used by rhizobia (Hood *et al*., 2015). However, in that case the difference in fixation phenotype was observed between plants that all belong to the galegoid tribe (e.g. *P. sativum* and *V. faba*). This raises an important caveat about our observation that Mn^2+^ transport by SitABCD and MntH is important for N_2_ fixation in galegoid legumes versus the phaseolid *P. vulgaris*. The absence of a fixation phenotype in *P. vulgaris* may be specific to this plant and independent of nodule development. However, the absence of an effect on N_2_ fixation when Mn^2+^ transport is prevented in both soybean and *P. vulgaris* suggests that nodule development may be important. In future it would be interesting to extend this by testing other host of *R. leguminosarum* including lentil (*Lens culinaris*). Fundamentally, these studies highlight the stark differences between nodules of different legumes and consequently, alter what is required by rhizobia for successful colonisation.

## Experimental procedures

### Bacterial strains, plasmids and culture conditions

The strains and plasmids used in this study are detailed in Table 1. *R. leguminosarum* strains were grown at 28°C in either tryptone‐yeast (TY) extract (Beringer, 1974) or Acid Minimal Salts (AMS) supplemented with 10 mM glucose and 10 mM NH_4_Cl (Poole *et al*., 1994). When growing the *sitA mntH* double mutant, solid TY medium was supplemented with 50 µM MnSO_4_. *Escherichia coli* strains were grown at 37°C in Luria Bertani (LB) broth or on LB‐agar. Antibiotics were used at the following concentrations (µg/ml^−1^): neomycin, 80; spectinomycin, 100 (50 for *E. coli*); streptomycin, 500; tetracycline, 2.

### Mutagenesis

Mutagenesis of *sitA* (RL3884) was achieved by amplifying the internal gene fragment with primers pr0970 and pr0971 and cloning the resulting product into XbaI‐digested pK19mob (pLMB452). Plasmid pLMB452 was then conjugated into Rlv38541 and site‐directed integration of the recombinant suicide vector was selected for with Neomycin as previously described (Karunakaran *et al*., 2009). The position of insertion was mapped using the primer pr0416 and the pK19mob mapping primer pK19/18B. Insertion in *sitA* was chosen as it is first gene in the operon and we expected it to completely disrupt transport by the Sit complex. All primers are shown in Supporting Information Table S1. Cloning utilised the BD In‐Fusion^TM^ cloning kit (Clontech) and was performed according to the manufacturer's instructions.

The gene *mntH* (RL0940) was disrupted with an omega intersposon cassette carrying Spc^r^ (ΩSpc). A 3 kb fragment containing *mntH* was PCR‐amplified from Rlv3841 genomic DNA using primers pr1186 and pr1187 and the resulting product was cloned into pJET1.2/blunt (pLMB543). A *Sma*I‐cut fragment carrying the ΩSpc cassette was cloned into pLMB543 at the unique EcoRV site in *mntH* (*mntH*ΩSpc) making pLMB544. A 5 kb XbaI/XhoI fragment the carrying *mntH*ΩSpc was then cloned into pJQ200SK to make pLMB546. The plasmid pLMB546 was conjugated into Rlv3841 and the single *mntH* mutant (LMB460) was isolated using the *sacB* mutagenesis strategy as described (Kumar *et al*., 2005). Mutagenesis was confirmed using intersposon primers (pOT forward/pOT forward_far) and mapping primers designed to bind ∼1 kb downstream and upstream of *mntH* (pr1225 and pr1226). To make the double mutant (LMB466), *mntH*ΩSpc was transduced from LMB460 into the single *sitA* mutant (LMB364) using the bacteriophage RL38 (Buchanan‐Wollaston, 1979). Solid TY medium supplemented with 50 µM MnSO_4_ was used when selecting for the double mutant.

To construct the single *sitA* and *mntH* mutations in RlvA34, *sitA*:pK19mob and *mntH*ΩSpc were transduced from LMB364 and LMB460 (respectively) into RlvA34, resulting in LMB525 and LMB526. The double mutant (LMB539) was made by transducing *sitA*:pK19mob from LMB364 into LMB526. RL38 is incapable of infecting Rlp4292 so the single and double mutations were created by *sac* mutagenesis. The plasmid pLMB546 was conjugated into Rlp4292 to make the single *mntH* mutant (LMB541). To make a single *sitA* mutant (LMB624), a 3 kb region containing *sitA* was PCR‐amplified from Rlp4292 genomic DNA (using primers pr1378 and pr1394) and cloned into pJET/1.2 blunt (pLM679). An *Eco*RI‐fragment carrying the intersposon cassette ΩKm was end‐filled using Klenow and then cloned into pLMB679 at the unique *Sma*I site in *sitA* resulting in pLMB691. An XbaI/NotI fragment from pLMB691 was then cloned into pJQ200SK making pLMB694, which was used to generate the single *sitA* mutation in Rlp4292. Mutagenesis was confirmed using intersposon primers (pOT forward/pOT forward_far) and mapping primers designed to bind ∼1 kb downstream and upstream of *sitA* (pr0416 and pr1457). To make the double mutation, pLMB694 was conjugated into LMB541 and the double mutant (LMB630) was isolated by *sac* mutagenesis on MnSO_4_‐supplemented TY medium.

### Complementation of the double *sitA mntH* mutant with pJP2*mntH*

To complement the double *sitA mntH* mutant with *mntH*, a 1.9 kb region containing *mntH* was amplified from Rlv3841 genomic DNA using primers pr1290 and pr1462. The PCR product was digested with XbaI/HindIII and cloned into XbaI/HindIII‐digested pJP2, to make pLMB766. The plasmid pLMB766 was then conjugated into the *sitA mntH* double mutant to make LMB683. Presence of the plasmid pLMB766 was confirmed with pJP2 mapping primers p611 and pr0096. To complement the *sitA* deletion in the double *sitA mntH* mutant, we constructed two plasmids (i) pOPS0393 (*sitA* along with promoter cloned in pJP2) and (ii) pOPS0394 (*sitABCD* operon along with promoter cloned in pJP2). Although, the pK19 insertion was only in *sitA*, we also complemented with *sitABCD* along with its promoter, to determine whether the pK19 insertion was polar on the rest of the operon. To construct pOPS0393 a 1.44 kb region containing *sitA* along with its native promoter was amplified from Rlv3841 genomic DNA using primers oxp1221 and oxp1220. The PCR product was digested with XbaI/HindIII and cloned into XbaI/HindIII‐digested pJP2. Similarly, to construct pOPS0394 a 4.0 kb region containing *sitABCD* along with its native promoter was amplified from Rlv3841 genomic DNA using primers oxp1221 and oxp1222. The PCR product was digested with XbaI/HindIII and cloned into XbaI/HindIII‐digested pJP2. The plasmids were then conjugated into the *sitA mntH* double mutant to make OPS0925 and OPS0926 respectively.

### Construction of *gusA*‐fusions and measurement of β‐glucuronidase (GUS) activity

For the construction of the *sitA*p‐*gusA* and *mntH*p‐*gusA* reporter fusions, the promoter‐regions were PCR‐amplified from Rlv3841 genomic DNA with primers pr1292 and pr1293 for *sitA* and pr1290 and pr1291 for *mntH* and cloned into pJP2 at the XbaI/HindIII sites to make plasmids pLB597 (*sitA*p‐*gusA*) and pLMB600 (*mntH*p‐*gusA*). Plasmids pLMB597 and pLMB600 were then conjugated into Rlv3841 to make LMB498 and LMB505 respectively. The presence of the plasmids was confirmed with pJP2 mapping primers p611 and pr0096.

To detect expression of *sitA*p‐*gusA* and *mntH*p‐*gusA in planta*, nodules taken from plants three weeks post inoculation were sectioned with a vibratome and incubated in staining buffer containing 0.02% 5‐bromo‐4‐chloro3‐inodyl‐β‐D‐glucuronide (Lodwig *et al*., 2004). After eighteen minutes, nodule sections were fixed in 1.25% glutaraldehyde and visualised under a Leica DM6000 light microscope.

When detecting expression of *sitA*p‐*gusA* and *mntH*p‐*gusA* in free‐living cells in response to MnSO_4_‐levels, strains were grown to an OD_600_ 1–1.2 in modified AMS glucose containing either 0.05 µM or 0.9 µM MnSO_4_. Cultures were sampled and β‐glucuronidase (GUS) activity measured as described (Lodwig *et al*., 2004). When detecting expression of *sitA*p‐*gusA* and *mntH*p‐*gusA* in free‐living cells in response to oxidative stress, cells were cultured in AMS glucose to an OD_600_ 0.2–0.4. The culture was split, where 100 µM H_2_O_2_ was added to one and 0 µM was added to the other. Samples were taken at 0, 2 4 and 6 h and used to measure GUS activity.

### Growth assays

Growth of Rlv3841, single and double mutants was tested in 96‐well plates. Strains were pre‐cultured in AMS or modified AMS glucose (containing 25 µM MnSO_4_) in the case of the double mutant, to an OD_600_ 0.2–0.6. Cultures were then split, spun down and resuspended in modified AMS (omitting MnSO_4_). This washing step was repeated twice to remove extracellular traces of MnSO_4_. After the final‐washing step, strains were suspended in modified AMS glucose containing 0.05 µM or 25 µM MnSO_4_ to an OD_600_ 0.1. Samples were then transferred to a 96‐well plate and measured at OD_600_ by a BioTek EON^TM^ plate reader. Growth was measured for 24 h at 30 min intervals, with linear shaking.

Growth of Rlv3841, RlvA34, Rlp4292 and their corresponding double mutants were tested in conical flasks. Strains were first grown on TY slopes or in the case of the double mutants, on TY supplemented with 50 µM MnSO_4_. After two days, slopes were washed with 5 ml modified AMS (omitting MnSO_4_) to obtain a bacterial suspension that was used inoculate modified AMS glucose containing 0.05 µM or 10 µM MnSO_4_ to a starting ∼OD_600_ 0.005. After 14 h of growth, samples were taken every 3–4 h and used to measure OD_600_. Note that 25 µM MnSO_4_ (used in the 96‐well plate assays) was inhibitory to Rlv3841 when cultured in conical flasks, hence 10 µM MnSO_4_ was used to restore growth of the double mutant in this assay.

### H_2_O_2_ sensitivity assay

To measure H_2_O_2_‐sensitivity, strains were first pre‐cultured in AMS glucose to stationary phase (OD_600_ 0.9–1.1). Cultures were then pelleted by centrifugation, washed three times in modified AMS (omitting MnSO_4_) and diluted to an OD_600_ 0.1. Diluted cultures were split, and 0.5 mM H_2_O_2_ was added to one and 0 mM to the other. Samples were taken after 0, 2, 4 and 6 h, serially diluted and then spotted onto solid AMS glucose medium. After two days’ growth, colony‐forming units (cfu)/ml for each sample was determined. When the *sitA mntH* double mutant was included, all strains were pre‐cultured in modified AMS glucose containing 25 µM MnSO_4_.

### Plant experiments

Seeds of *P. sativum* cv. Avola and scarified *V. faba* cv. Sutton were surfaced sterilised (30 secs 70% EtOH and 5 mins 2% sodium hypochlorite), washed extensively with sterile distilled water and sown into in 1 L pots containing autoclaved vermiculite and 400 ml N_2_‐free rooting solution (Poole et al., 1994). *P. vulgaris* cv. Tendergreen seed were treated and sown using the same method, with the exception of shorter washing steps (only leaving seeds in sterile H_2_O for 5 seconds). *V. hirusta* seeds were scarified and surface sterilised with 1% sodium hypochlorite (5 mins) before washing with sterile distilled water. These seeds were then placed onto 3% w/v H_2_O‐agar and kept in the dark to germinate. After two days, germinated seeds were sown into 1 L pots containing autoclaved vermiculite and N_2_‐free rooting solution. Plants were inoculated with 10^6^ cfu of *R. leguminosarum*, grown in a controlled growth room at 22°C with a 16 h light/8 h dark cycle and then harvested three weeks post inoculation. Acetylene reduction of plants was determined in 95% air‐5% acetylene for 1 h in 250 ml Schott bottles as described (Hardy *et al*., 1973; Trinick *et al*., 1976).

Root nodules were sectioned and then stained with toluidine blue. Stained sections were visualised under a Leica DM6000 light microscope. For electron microscopy, ultrathin sections were taken and stained with uranyl acetate and lead citrate as previously described (Lodwig *et al*., 2003).

## Supporting information

Additional Supporting Information may be found in the online version of this article at the publisher's web‐site:

**Fig. S1.** Growth curves of the double *sitA mntH* mutant complemented with pJP2(sitA) or pJP2(sitABCD). Rlv3841 (diamonds), double mutant *sitA mntH* (triangle), double mutant complemented with pJP2(*sitA*) and double mutant complemented with pJP2(*sitABCD*) were grown in media limited (0.05 µM) or not limited (25 µM) for MnSO_4_. Averaged from three independent experiments. For clarity only plus SEM bars are shown at 4 h intervals.

**Fig. S2.** GusA activity in strains expressing *sitAp*‐*gusA* and *mntHp*‐*gusA* in response to H_2_O_2_ and in absence of OxyR. GUS activity in Rlv3841 and the *oxyR* mutant carrying either *sitAp*‐*gusA* (a) or *mntHp*‐*gusA* (b). Measured in the absence or presence of H_2_O_2_ (100 µM). Averaged from three independent experiments ± SEM.

**Table S1**. Primers used in this study.
