# Supplementary figures and images for: Manganese transport is essential for N2‐fixation by Rhizobium leguminosarum in bacteroids from galegoid but not phaseoloid nodules
Source: Environ Microbiol. 2017 May 30;19(7):2715–26. doi: 10.1111/1462-2920.13773 (PMC5575495; doi:10.1111/1462-2920.13773)

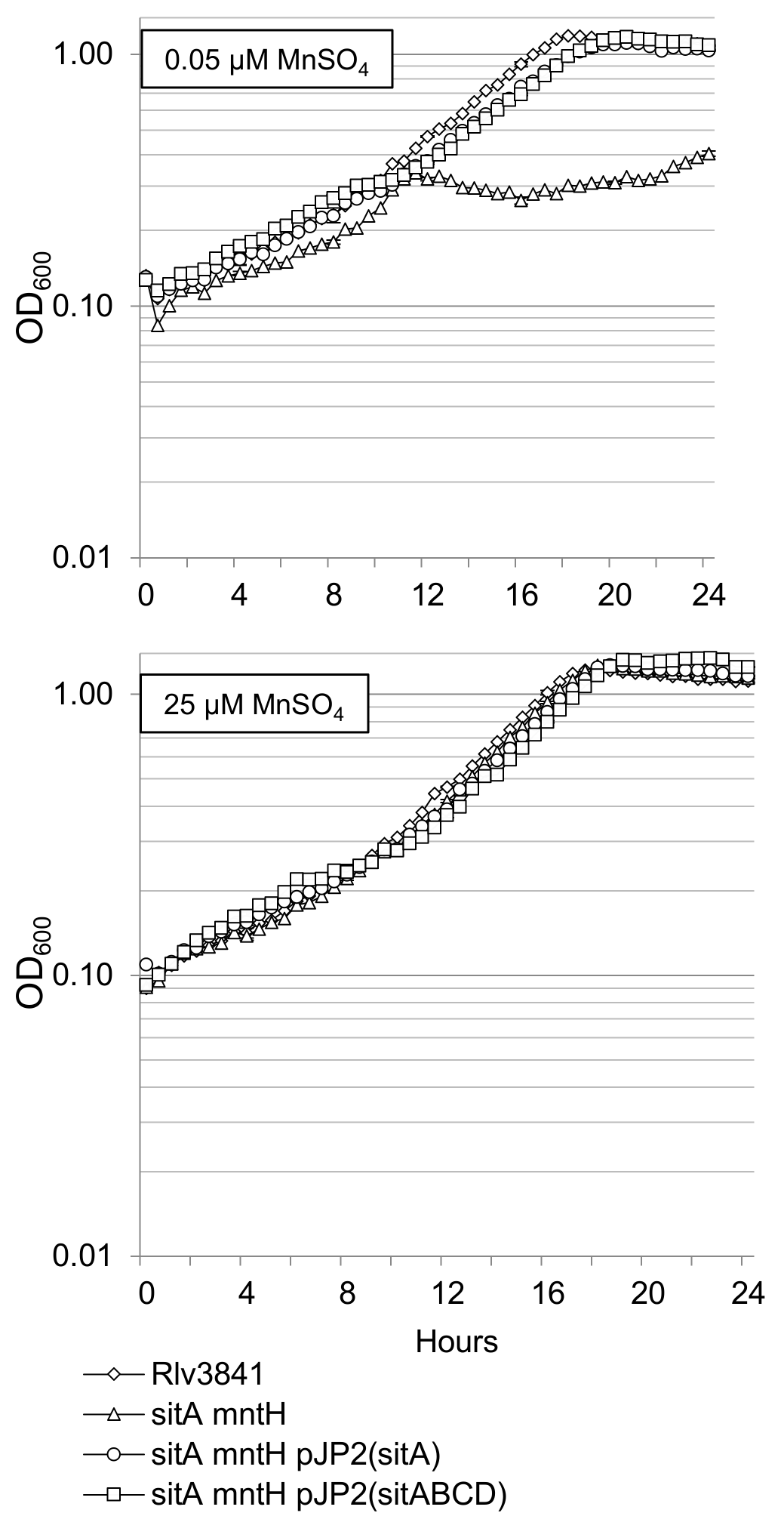

Supplement: Supplementary file 1 — Fig. S1. Growth curves of the double sitA mntH mutant complemented with pJP2(sitA) or pJP2(sitABCD). Rlv3841 (diamonds), double mutant sitA mntH (triangle), double mutant complemented with pJP2(sitA) and double mutant complemented with pJP2(sitABCD) were grown in media limited (0.05 µM) or not limited (25 µM) for MnSO4. Averaged from three independent experiments. For clarity only plus SEM bars are shown at 4 h intervals. [file EMI-19-2715-s001.tiff]

a. GUS activity for *sitA-gusA*

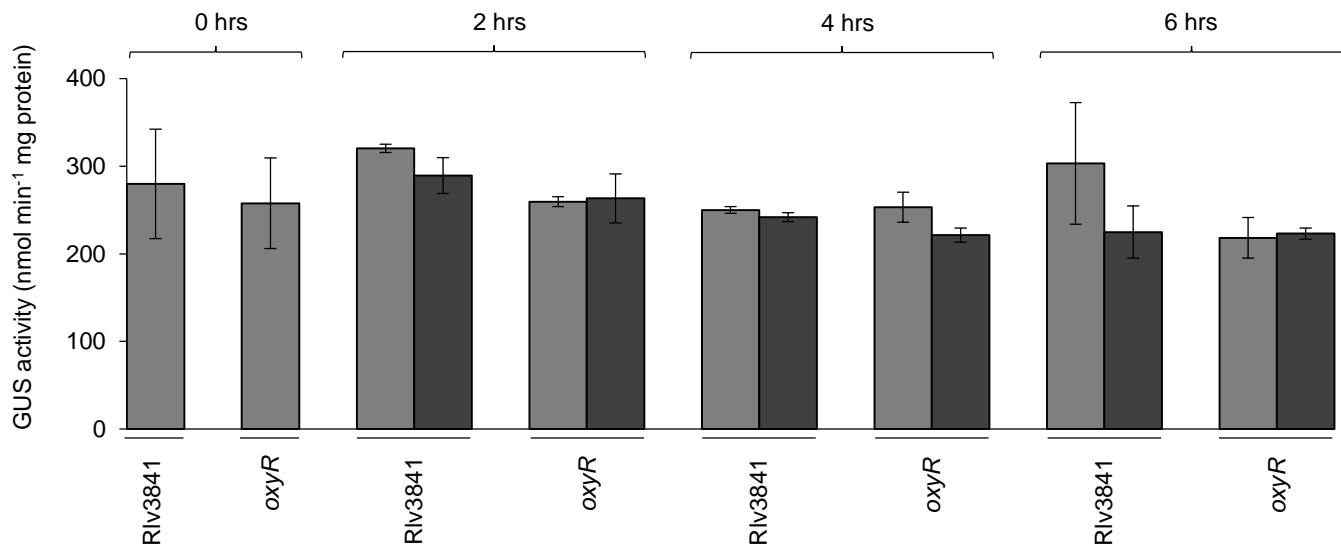

b. GUS activity for *mntH-gusA*

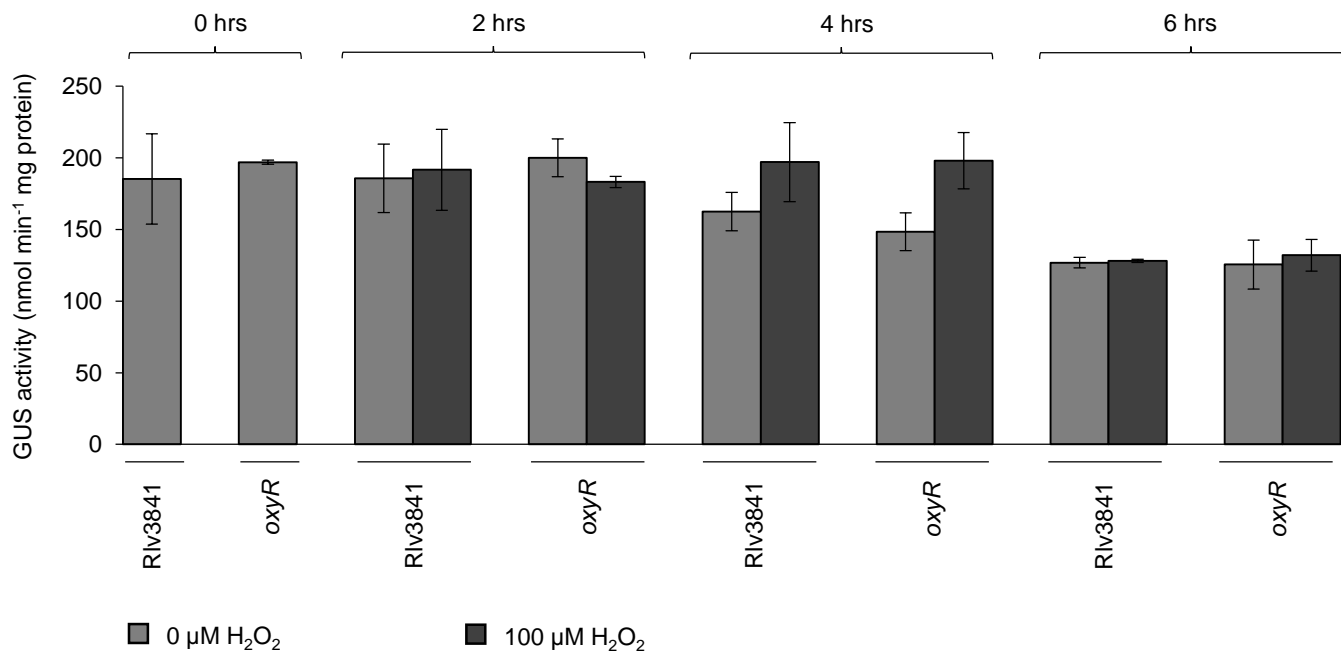

Supplement: Supplementary file 2 — Fig. S2. GusA activity in strains expressing sitAp‐gusA and mntHp‐gusA in response to H2O2 and in absence of OxyR. GUS activity in Rlv3841 and the oxyR mutant carrying either sitAp‐gusA (a) or mntHp‐gusA (b). Measured in the absence or presence of H2O2 (100 µM). Averaged from three independent experiments ± SEM. [file EMI-19-2715-s002.pdf]
