# Supplementary material for: Manganese transport is essential for N2‐fixation by Rhizobium leguminosarum in bacteroids from galegoid but not phaseoloid nodules
Source: Environ Microbiol. 2017 May 30;19(7):2715–26. doi: 10.1111/1462-2920.13773 (PMC5575495; doi:10.1111/1462-2920.13773)
Supplement: Supplementary file 3 — Table S1. Primers used in this study. [file EMI-19-2715-s003.docx]

**Table S1. Primers used in this study**

| **Primers** | **Sequence** | **Description** |
| --- | --- | --- |
| M13uni (-21) | TGTAAAACGACGGCCAGT | Mapping/sequencing primer; pK19mob and pRU877 |
| M13rev (-29) | CAGGAAACAGCTATGACC | Mapping/sequencing primer; pK19mob and pRU877 |
| pK19/18B | GCACGAGGGAGCTTCCAGGG | Mapping primer for pK19mob integration |
| pOT forward | CGGTTTACAAGCATAAAGC | Mapping primer; intersposon mutagenesis |
| pOT forward_far | GACCTTTTGAATGACCTTTA | Mapping primer; intersposon mutagenesis |
| p611 | GCGATCCAGACTGAATGCCC | Mapping/sequencing primer; pJP2 |
| pr0096 | TCGTAAATGCTGGACCCGATGG | Mapping/sequencing primer; pJP2 |
| pr0970 | GCAGGTCGACTCTAGAACAGACAACCAATTCGAAGT | Forward primer; BD cloning of *sitA* (RL3884) |
| pr0971 | CCGGGGATCCTCTAGATAAAGCACGCCTCCATAGTG | Reverse primer; BD cloning of *sitA* (RL3884) |
| pr0416 | GGGACGGACAAGATTGCC | Mapping primer for *sitA* (RL3884) mutagenesis |
| pr1186 | CGTATAGACGCGGCGTTCGA | Forward primer; *mntH* (RL0940) |
| pr1187 | AGGGCATGAGCGTGCTGGAA | Reverse primer; *mntH* (RL0940) |
| pr1225 | GCAGCACCTTCGAGCGAGAC | Mapping primer for *mntH* (RL0940) mutagenesis |
| pr1226 | CCTTAGACAGAATGAGCTGG | Mapping primer for *mntH* (RL0940) mutagenesis |
| pr1378 | CGAGCTTTCCGGCGGCCAGA | Forward primer; Rlp4292 *sitA* |
| pr1394 | GCGTCACCGCCGTCGTCGGC | Reverse primer; Rlp4292 *sitA* |
| pr1457 | CGTTGAGCTGATCGACCATG | Mapping primer for Rlp4292 *sitA* mutagenesis |
| pr1292 | AAGCTTCCTATCTGGTCTTCAAGGCC | Forward primer; *sitA* (RL3884) promoter |
| pr1293 | TCTAGATTGGTTGTCTGTTGGGCAGC | Reverse primer; *sitA* (RL3884) promoter |
| pr1290 | AAGCTTTCAGGCGCGACTGGACGGGC | Forward primer; *mntH* (RL0940) promoter |
| pr1291 | TCTAGATCGCCATGCCGAGCTGTGAC | Reverse primer; *mntH* (RL0940) promoter |
| pr1462 | TCTAGAGCTGCGTGCGCCTCTCGTCA | Reverse primer; *mntH* (RL0940) |
| oxp1220 | TTTTTCTAGAGCATCCTTTACCGTGATCCC | Reverse primer bind to 3’ end of *sitA* with XbaI site |
| oxp1221 | TTTTAAGCTTCCTATCTGGTCTTCAAGGCCT | Forward primer to 5’ *sitA* promoter with HindIII |
| oxp1222 | TTTTTCTAGATTAGGCAGCAGCTCAAATTGT | Reverse primer to 3’ end of *sitD*, to amplify *sitABCD* operon with XbaI |
